# Comparison of 2 femoral tunnel drilling techniques in anterior cruciate ligament reconstruction. A prospective randomized comparative study.

**DOI:** 10.1186/s12891-018-2376-0

**Published:** 2018-12-22

**Authors:** Yunhang Geng, Pengzhou Gai

**Affiliations:** 1 Qingdao University Medical College, Qingdao, China; 2 Department of Orthopaedic Surgery, Qindao University Medical College Affiliated Yantai Yuhuangding Hospital, Yantai, Shandong People’s Republic of China

**Keywords:** Anterior cruciate ligament, Anteromedial portal, Transtibial, Femoral tunnel

## Abstract

**Background:**

To evaluate the length and position of femoral tunnel,and exam whether knee stability and clinical functional outcomes are superior in AMP method.

**Methods:**

From August 2014 to February 2015, we prospectively recruited 104 patients undergoing anterior cruciate ligament reconstruction. They were randomized to anteromedial portal or transtibial method. All patients underwent Lysholm score, International Knee Documentation Committee score,Tegner score at pre-operative and last follow-up point as subjective assessment of clinical function. The Lachman test, the Pivot-shift test and KT-1000 were performed at the last follow-up as a evaluation of knee joint stability. We measured the length of femoral tunnel intraoperatively and at 1 week post-operatively, CT-based three-dimensional reconstruction was used to assess femoral tunnel location.

**Results:**

The average follow-up time of anteromedial portal group was 25.7 ± 6.8 months (range:12–36.5 months), and the average follow-up time of the transtibial group was 24.9 ± 6.0 months (range:12–37 months). There was no significant difference between the groups pre-operative Lysholm score, IKDC score and Tegner scores. Both groups showed significantly improvement in these clinical function scores at follow up for their ACL reconstruction. However, there was no significant difference in the function scores between the two groups at last follow up. However, the mean femoral tunnel length in the anteromedial portal group was significantly shorter than that in the transtibial group. And tunnel location was significantly lower and deeper with the anteromedial portal technique than with the transtibial technique.

**Conclusion:**

The use of anteromedial portal method resulted in a significantly lower and deeper placement of the femoral tunnel, and a shorter tunnel length compared to the transtibial method. However, there was no statistical difference in terms of clinical function and knee joint stability between the anteromedial portal method and the transtibial method.

**Trial registration:**

Name of the registry: Chinese Clinical Trial Registry.

The registration number: ChiCTR1800014874.

The date of registration: 12 February, 2018.

The study is retrospectively registered.

## Background

The anterior cruciate ligament (ACL) is an important static stabilizer of the knee joint and plays an important role in both anteroposterior and rotational stability [1]. Injury seriously affects this stability, requiring surgical reconstruction as the main method for restoring knee stability and function. The transtibial (TT) method is widely used for placing the femoral tunnel integral to ACL reconstruction. However, this approach can limit the accuracy of femoral tunnel placement, and failure of the reconstruction may be influenced most by inaccurate tunnel placement [2, 3]. The anteromedial portal (AMP) approach is becoming more popular as femoral tunnel placement is not limited by the tibial tunnel, resulting in easier and potentially more accurate location of the femoral tunnel in the ACL anatomical footprint [4–7]. Differences have been shown in femoral tunnel location between AMP and TT methods in previous retrospective studies [5, 8–10]. And Gadikota HR et al. [11] used a corpse-controlled study, and findings of this study indicated that a larger posterolateral bundle coverage was achieved by the AM portal than by the TT technique and center of the tunnel created by the AM portal was closer to the native ACL footprint center than the center of the TT technique tunnel. In addition to, meta-analysis of Riboh JC et al. [12] also demonstrated that more anatomic graft placement with AMP method.

Although graft placement with AMP method is more anatomic,the length of the femoral tunnel is statistically significantly shorter with the AMP approach compared to TT method [5, 8, 9, 13]. A shorter femoral tunnel (less than 25 mm) makes the femoral tendon - bone effective healing length shorter, and leading to the smaller the healing strength [14].

Nowadays, there is still controversy about the stability of the knee joint between two methods. Musahl V et al. [15] used a corpse-controlled study, and findings of this study indicated that knee kinematics was closer to the intact knee with the AMP technique. The prospective randomized comparative study of Hussein M et al. [16] demonstrated that anteroposterior and rotational stability of the knee was better with the AMP technique. The meta-analysis of Chen H et al. [17] and Riboh JC et al. [12] both demonstrated that with the AMP technique was better than that with the TT technique in terms of postoperative stability. However,retrospective study of Rezazadeh S et al. [1] demonstrated that anteroposterior and rotational stability did not differ statistically between two methods. And, the prospective randomized comparative study of Bohn MB et al. [18] demonstrated that no significant difference in rotational stability walking, running, and pivoting was seen between two techniques at 1-year follow-up.

Similarly, there is still controversy about the function of the knee between two methods. Previous retrospective studies [1, 6, 7, 19, 20] have not definitively demonstrated a resultant clinical superiority in knee function with AMP technique. And, the prospective randomized comparative study of Hussein M et al. [16] and meta-analysis of Riboh JC et al. [12] both demonstrated that there was no statistical difference between the two groups on knee function. However,the retrospective cohort study of Kilinc BE et al. [21] demonstrated that ACL reconstruction with AMP method was better than with the TT technique in term of clinical function. The meta-analysis of Chen H et al. [17] also demonstrated that with the AMP technique is better than that with the TT technique in terms of functional recovery of the knee.

Our research is to evaluate the length and position of femoral tunnel,and exam whether knee stability and clinical functional outcomes are superior in AMP method.

## Methods

### Patients

From August 2014 to February 2015, we prospectively recruited patients undergoing autologous hamstring tendon ACL reconstruction. ACL damage was diagnosed by clinical manifestations and MRI (GE3T) findings. Inclusion criteria were at least 1 year post-operative follow up, normal contralateral knee joint, no accompanying fracture. Exclusion criteria were combined anterior and posterior cruciate ligament injury, accompanying collateral ligament injury, osteoarthritis and revision surgery. Prior to the study, according to the literature and our own experience, it was calculated that there must be 48 patients in each group when we accepted the difference in tunnel location between groups being 2% (assuming a standard deviation of 3.5%, at 80% power and **α** error of 0.05). According to our own experience, considering almost 20% rate of lost of follow up, a total of 120 patients were required for the study. Randomisation was performed according to a random number table, with even numbers in the AMP group and odd numbers in the TT group. Initially there were 63 patients in AMP group and 57 patients in TT group. Because of lost of follow-up, 7 patients in AMP group were excluded and 9 patients in TT group were excluded. Finally, 56 were included in AMP group and 48 were included in TT group. The population assessed is predominantly male. This study was approved by the Qindao University Medical College Affiliated Yantai Yuhuangding Hospital Ethics Committee (Numbered:YHY2014065), and all patients signed informed consent. This study has been registered in Chinese Clinical Trial Registry (Numbered: ChiCTR1800014874. URL: http://www.chictr.org.cn/showproj.aspx?proj=25277. UTN: U1111–1210-8275 Registrant: Pengzhou Gai).

### Surgical procedure

All operations were performed under general anesthesia or epidural anesthesia by the same orthopaedic surgeon. Arthroscopy was used to further confirm the diagnosis by conventional anteromedial and anterolateral portals. The ruptured ACL was cleaned using a planer. Contours of the intercondylar notch were observed with knee extension at 30 °. If the intercondylar notch was narrow, it was expanded with 5.5 mm drill to an inverted “U” shape, although this was not commonly required in our cohort. A medial longitudinal incision over the tibial tubercle was performed to harvest Semitendinosus and Gracilis tendon, which were dissected, woven, preprocessed and reserved. The tibial tunnel locator was placed with the outer end located at least 3 cm below the joint line and 1.5 cm medial to the tibial tubercle (inside the tendon incision), and the inner end located at the center of insertion of the anterior cruciate ligament. The guide pin was inserted, and the cannulated reamer was selected according to the graft diameter. Then the tunnel was drilled along the guide pin.

**Fig. 1:**
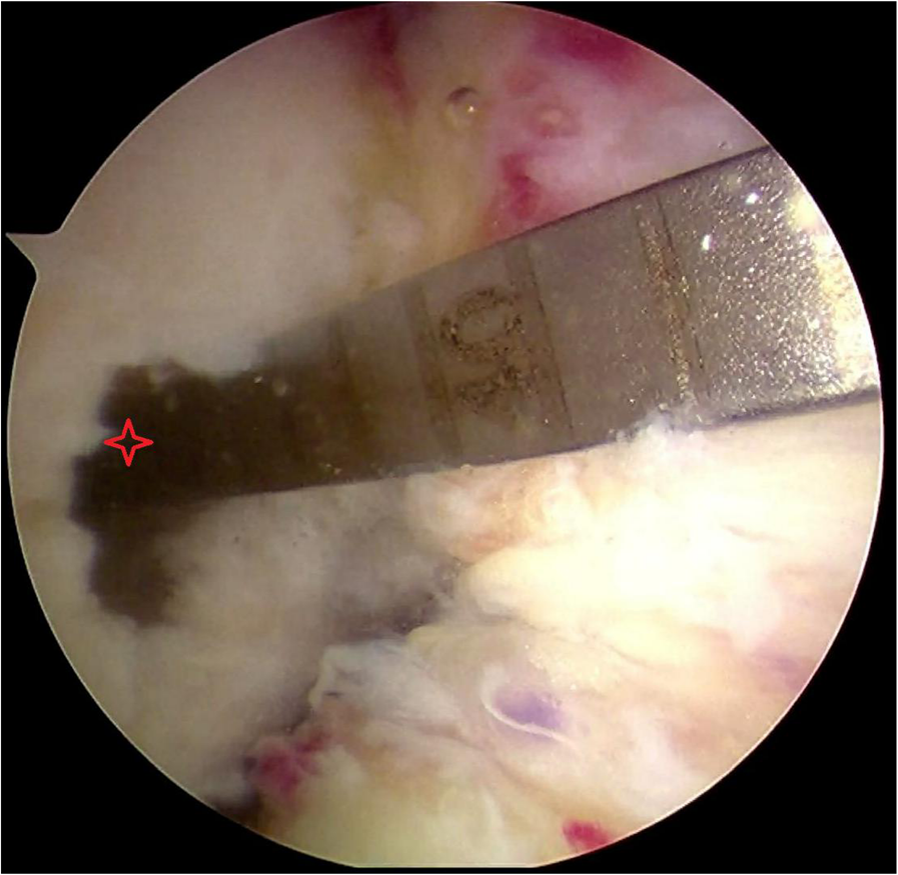
A depth gauge was used to hook the outer cortex of the femoral tunnel and intraoperative measurement of femoral tunnel depth is seen(*)

For TT method the femoral tunnel locator was placed via tibial tunnel under arthroscopy and for AMP method via anteromedial portal under arthroscopy. The posterior wall of the femoral tunnel is at least 2 mm from the posterior cortex of the femoral condyle. A guide pin is then placed and cannulated reamer selected according to the graft diameter. Tunnel depth of drilling is approximately 2.1 cm, then beyond this a 0.45 cm cannulated reamer is used. A depth gauge (Smith & Nephew) was used to hook the outer cortex of the femoral tunnel and total length of the tunnel recorded (Fig. 1). Grafts were transferred to the tunnel through wire and suture. Suitable Endobutton (Smith & Nephew) was used based on the length of the femoral tunnel. Intrafix (Depuy) was used according to the diameter of the tibial tunnel. Finally the ligament isometry and the intercondylar notch impingement was checked.

### Evaluation methods

**Fig. 2:**
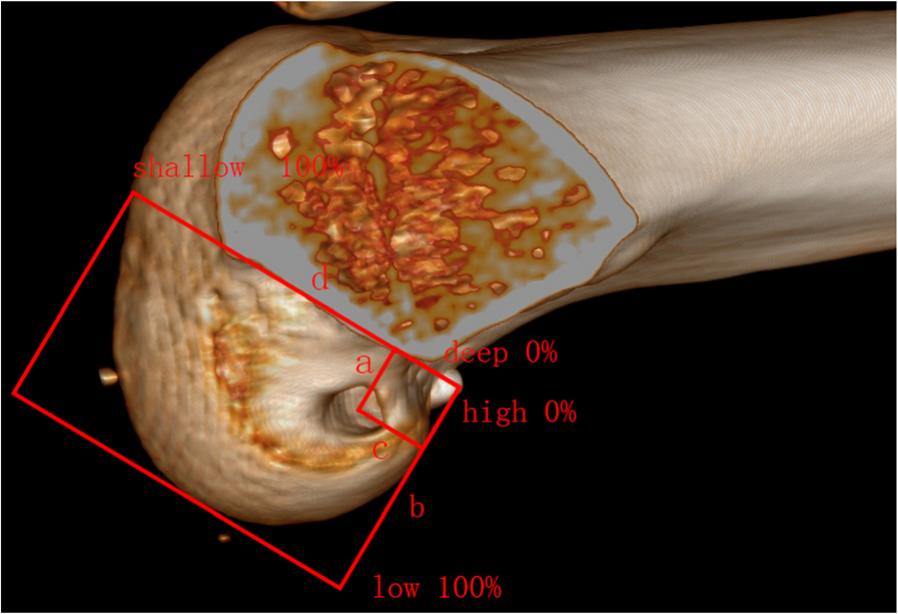
Blumensaat’s line is represented by ‘d’,with ‘b’ representing the line perpendicular to it. ‘a’ is the distance between the femoral tunnel center to Blumensaat’s line, and ‘c’ is the distance between the femoral tunnel center to b. Thus, a/b represents the height ratio, i.e., the ratio of the distance between the femoral tunnel center and the top of intercondylar notch to the total height of intercondylar notch; and c/d represents the depth ratio, i.e., the ratio of the distance between the femoral tunnel center and posterior articular surface of femoral lateral condyle to the total depth of intercondylar notch

All evaluations were performed by one doctor who was blind to the treatment group. All patients underwent Lysholm score, International Knee Documentation Committee (IKDC) score and Tegner score at pre-operative and final follow-up points as subjective assessment of clinical function. The Lachman test, the Pivot-shift test and KT-1000 (MEDmetric Corporation, San Diego, California) were performed at the last follow-up as a evaluation of knee joint stability. We measured the length of femoral tunnel intraoperatively. And after 1 week post-operatively, all patients were performed CT-based three-dimensional reconstruction (GE64 CT AW4.3 workstation, MIMICS) to assess the location of the femoral tunnel. Measurement of femoral tunnel location used the quadrant method described by Bernard M et al. [22] (Fig. 2).

### Statistical analysis

SPSS 20.0 (SPSS Inc., Chicago, IL) software and G*Power (3.1.9.2) software was used for statistical analysis. To compare age, follow-up time, time from injury to surgery, body mass index (BMI), Lysholm score, IKDC score, Tegner score, KT-1000, tunnel length and tunnel location, unpaired T test was used. For gender and injury side of the knee, chi-square test was selected, and Mann-Whitney rank test for the Lachman and Pivot-shift test comparisons. Statistical significance was at *P* < 0.05.

## Results

The average follow-up time of anteromedial portal group was 25.7 ± 6.8 months (range:12–36.5 months), and the average follow-up time of the transtibial group was 24.9 ± 6.0 months (range:12–37 months). 24 underwent notchplasty in AMP group and 17 underwent notchplasty in TT group.

General demographic data were not significantly different between the two groups (Table 1).

**Table 1:** General Information

Variate	AMP group	TT group	*P* value
Patient(n)	56	48	
Gender (male:female,n)	46:10	40:8	.873
Age (Mean ± SD, range, year)	29.6 ± 11.7 (18–65)	31.8 ± 11.0 (19–69)	.346
Follow-up time(Mean ± SD,range, months)	25.7 ± 6.8 (12–36.5)	24.9 ± 6.0 (12–37)	.529
Time from injury to surgery(Mean ± SD,range, weeks)	9.8 ± 6.3 (0.5–24)	10.6 ± 6.8 (0.5–28)	.557
Injured knee (left:right,n)	26:30	22:26	.952
BMI(Mean ± SD,range, kg/m^2^)	24.1 ± 3.2 (20–32)	23.8 ± 3.1 (18–31)	.636

*AMP*:Anteromedial portal, *TT*:Transtibial, *BMI*:Body mass index

There was no significant difference between the groups pre-operative Lysholm score, IKDC score and Tegner scores. Both groups showed significantly improvement in these clinical function scores at follow up for their ACL reconstruction. However, there was no significant difference in the function scores between the two groups at last follow up. (Table 2 and Table 3).

**Table 2:** Clinical function score between two groups

	Pre-operative			Last follow-up		
	AMP group	TT group	*P* value	AMP group	TT group	*P* value
Lysholm score (Mean ± SD,range)	45.9 ± 6.6(24–70)	45.4 ± 6.2(23–67)	.729	93.3 ± 5.0(75–97)	91.6 ± 6.5(68–97)	.123
IKDC score (Mean ± SD,range)	35.7 ± 6.3(15–55)	36.1 ± 6.6(18–55)	.703	89.5 ± 8.8(50–97)	87.4 ± 10.0(50–95)	.273
Tegner score (Mean ± SD,range)	5.0 ± 1.0(1–7)	5.0 ± 1.1 (1–6)	.778	6.8 ± 1.4 (2–8)	6.3 ± 1.4 (2–8)	.081

*AMP*:Anteromedial portal, *TT*:Transtibial, *IKDC*: International Knee Documentation Committee

**Table 3:** Clinical function score between pre-operative and post-operative

	AMP group			TT group		
	Pre-operative	Last follow-up	*P* value	Pre- operative	Last follow-up	*P* value
Lysholm score (Mean ± SD,range)	45.9 ± 6.6(24–70)	93.3 ± 5.0(75–97)	<.01	45.4 ± 6.2(23–67)	91.6 ± 6.5(68–97)	<.01
IKDC score (Mean ± SD,range)	35.7 ± 6.3(15–55)	89.5 ± 8.8 (50–97)	<.01	36.1 ± 6.6(18–55)	87.4 ± 10.0(50–95)	<.01
Tegner score (Mean ± SD,range)	5.0 ± 1.0(1–7)	6.8 ± 1.4 (2–8)	<.01	5.0 ± 1.1 (1–6)	6.3 ± 1.4 (2–8)	<.01

*AMP*:Anteromedial portal, *TT*:Transtibial *IKDC*: International Knee Documentation Committee

In terms of Lachman test, Pivot-shift test and KT-1000 for assessment of knee joint stability, there was no statistical difference at the last follow-up between the two groups (Table 4).

**Table 4:** Knee joint stability assessment

	AMP group(*n* = 56)	TT group(*n* = 48)	*P* value
Lachman test			.630
Negative	44	36	
1+	12	11	
2+	0	1	
3+	0	0	
Pivot-shift test			.146
Negative	44	32	
1+	12	14	
2+	0	2	
3+	0	0	
KT 1000 (Mean ± SD,range,mm)	1.5 ± 0.9 (1–4)	1.6 ± 0.8 (1–5)	.455

*AMP* Anteromedial portal, *TT* Transtibial

The mean femoral tunnel length in the AMP group was significantly shorter than that in the TT group. And tunnel location was significantly lower and deeper with the AMP technique than with the TT method (Table 5).

**Table 5:** Tunnel parameters

	AMP group	TT group	*P* value
Tunnel length (Mean ± SD,range,mm)	37.3 ± 3.9 (28–46)	42.0 ± 4.8 (32–55)	<.01
Tunnel position			
Depth ratio (Mean ± SD,range,%)	20.3 ± 2.0 (17–23)	22.1 ± 2.1 (19–26)	<.01
Height ratio (Mean ± SD,range,%)	33.3 ± 1.8 (30–36)	30.2 ± 1.7 (27–33)	<.01

*AMP*:Anteromedial portal, *TT*:Transtibial

## Discussion

Previous studies demonstrated that [5, 8–12] center of the tunnel created by the AM portal was closer to the native ACL footprint center than the center of the TT technique tunnel. And although graft placement with AMP method is more anatomic,the length of the femoral tunnel is statistically significantly shorter with the AMP approach compared to TT method [5, 8, 9, 13]. In addition to, controversy continues as to whether the AMP or TT method is superior for ACL reconstruction in terms of both stability and function of knee [1, 5–9, 12, 16–21]. The most important finding of our study was that the use of AMP method resulted in a significantly lower and deeper placement of the femoral tunnel, and a shorter tunnel length compared to the TT method. However, there was no statistical difference in terms of clinical function and knee joint stability between the AMP method and TT method.

In our study there was no statistically significant difference in the Lysholm score, the IKDC score and the Tegner score between the AMP group and TT group at last follow-up. Similarly,previous retrospective studies [1, 6, 7, 19, 20] have not definitively demonstrated a resultant clinical superiority in knee joint function with AMP technique. And, the prospective randomized comparative study of Hussein M et al. [16] and meta-analysis of Riboh JC et al. [12] both demonstrated that there is no statistical difference between the two groups on knee function. However,the retrospective cohort study of Kilinc BE et al. [21] demonstrated that ACL reconstruction with AMP method was better than with the TT technique in term of clinical function. The meta-analysis of Chen H et al. [17] also demonstrated that with the AM technique was better than that with the TT technique in terms of functional recovery of the knee.

We found no statistically significant difference in the Lachman test, the Pivot-shift test and the KT-1000 measurement between the AMP group and TT group. Similarly,the retrospective study of Rezazadeh S et al. [1] demonstrated that anteroposterior and rotational stability did not differ statistically between two methods. And,the prospective randomized comparative study of Bohn MB et al. [18] also demonstrated that no significant difference in rotational stability walking, running, and pivoting was seen between two techniques at 1-year follow-up. However, Musahl V et al. [15] used a corpse-controlled study, and demonstrated that knee kinematics was closer to the intact knee with the AMP technique. The prospective randomized comparative study of Hussein M et al. [16] also demonstrated that anteroposterior and rotational stability of the knee was better with the AMP technique. The meta-analysis of Chen H et al. [17] and Riboh JC et al. [12] both demonstrated that with the AM technique were better than that with the TT technique in terms of postoperative stability.

TT method has the advantages of a simpler operation with shorter operation time, small incision and parallel tibial-femoral tunnel allowing for easy graft passage [23]. However, the most important drawback is that due to the limitations of the tibial tunnel, it is difficult to accurately locate the femoral tunnel in the anatomical ACL footprint, and the femoral tunnel is often located near the top of the intercondylar notch [23]. This is deemed a major factor for failure of ACL reconstruction [2]. In addition with this method, the surgeon may compromise the tibial tunnel in order to achieve the required femoral tunnel location [23, 24]. This can then result in short tibial tunneling that affects the fixation of the graft at the tibial end [24]. The AMP method not only makes it easier to locate the femoral tunnel in the ACL anatomical footprint, it also enables a more oblique femoral tunnel [25], reduces tunnel widening and can help retain residual ACL fibers [23]. But the AMP approach can result in a shorter femoral tunnel [25] and difficulty passing graft material through the tunnel [23]. Technical requirements are high and there is a greater risk of posterior breach in the condylar cortex or damage to the posterior articular cartilage and common peroneal nerve [23, 26].

The length of the femoral tunnel measured in this study was statistically significantly shorter with the AMP approach compared to TT method. A shorter femoral tunnel (less than 25 mm) makes the femoral tendon - bone effective healing length shorter, and leading to the smaller the healing strength [14]. However, the results of this paper and Shin YS et al. [9] show that the mean length of femoral tunnel prepared by AMP method, although shorter, is still greater than 30 mm, and none of the femoral tunnel lengths were shorter than 25 mm. Larson AI et al. [8] used a corpse-controlled study, and their results also show AMP group average femoral tunnel length greater than 30 mm. Osti M et al. [5] also demonstrate an average femoral tunnel length above 30 mm but suggest caution, as the AMP group(*n* = 32) did have a higher, though not statistically significant proportion(6%) with a femoral tunnel shorter than 25 mm. The corpse-controlled study of Bedi A et al. [13] showed that 41.7% of the femoral tunnel lengths in the AMP group (*n* = 12) were shorter than 25 mm and with the knee flexed to 120 °, the mean length of the femoral tunnel prepared by the AMP method was 21.3 mm.

In this paper, the femoral tunnel location was evaluated using CT-based three-dimensional reconstruction and the quadrant method described by Bernard M et al. [22]. Tunnel location in our study was significantly a deeper, lower and more anatomical with the AMP technique than with the TT method. Similarly, Osti M et al. [5] although a non randomized study, showed that tunnel location was significantly lower with the AMP technique but there was no statistically significant difference in depth between the two methods. Larson AI et al. [8] and Gadikota HR et al. [11] used a corpse-controlled study, and both demonstrated the location of femoral tunnel was significantly lower and deeper. In addition to, the meta-analysis of Riboh JC et al. [12] also demonstrated that more anatomic graft placement with AMP method. The deeper and lower the femoral tunnel, the more inclined the graft, which not only maintains the anteroposterior stability, but also the rotational stability. The evidence suggests overall that the AMP approach better locates the femoral tunnel in an anatomical footprint [6, 7, 11, 12, 19], with a deeper and lower tunnel location that is potential superior for maintaining the stability of the knee. This should translate clinically into better outcomes on knee stability testing. However, our study was not able to demonstrate this difference to statistical significance in term of knee stability testing.

Our studies strengths include a prospective randomized design, with one surgeon performing the procedures to reduce operative variability and the use of CT-based three-dimensional reconstruction as a recognized and reliable method of assessing femoral tunnel location. Our study includes larger group sizes than the majority of other studies, and extends the evidence whether length and position of femoral tunnel, knee stability and clinical functional outcomes are superior in AMP method.

The limitations include a relatively short follow-up time, with a mean around 25 months. It is therefore not possible to comment on differences in stability or function that may or may not be apparent over a longer timeframe. Although larger than most studies on this topic, the sample size is still relatively small, and thus may not be able to demonstrate significant differences between the groups. In addition, as reflective of the population most affected by these types of ACL injury, the population assessed is young and predominantly male, without other concurrent injuries or osteoarthritis and therefore findings may not apply to those patients that fall outside of these characteristics. For future directions a prospective randomized trial with larger sample size and longer term follow up is required. An assessment of other aspects such as operative times, costs and complications including graft failure rates would also be informative in recommending a particular method.

Our current study demonstrated that the AMP approach provides a deeper, lower and more anatomically placed femoral tunnel location compared to TT method. AMP technique produces a shorter tunnel, which surgeons performing this procedure should be mindful of. Those undergoing AMP repair were not proven statistically significant in term of function and stability of knee, and as such the AMP approach cannot be conclusively declared the superior method.

## Conclusion

The use of AMP method resulted in a significantly lower and deeper placement of the femoral tunnel, and a shorter tunnel length compared to the TT technique. However, there was no statistical difference in terms of clinical function and knee joint stability between the AMP method and TT method.

## Abbreviations

ACL: Anterior cruciate ligament
AMP: Anteromedial portal
BMI: Body mass index
IKDC: International Knee Documentation Committee
TT: Transtibial
